# Biochemical Studies on the Therapeutic Effect of *Naja nubiae* Venom Against Melamine Induced Hepatotoxicity in Albino Rats

**DOI:** 10.1002/fsn3.70081

**Published:** 2025-04-07

**Authors:** Al‐Shimaa M. Abas, Akaber T. Keshta, Samah A. Mohammed, Shimaa H. Watad

**Affiliations:** ^1^ Biochemistry Department, Faculty of Science Zagazig University Zagazig Egypt

**Keywords:** cobra venom, hepatotoxicity, IL‐10, INF‐γ, melamine

## Abstract

Illegal melamine analogs are added to food to make it appear as though it contains more protein. These substances have negative impacts on both humans and animals in high quantities. The present paper examines how cobra venom shields rats from melamine‐induced hepatotoxicity. The current study was conducted on six groups of adult male rats, as follows: group I (negative control): I.P. injected with distilled water, group II (SV10 μg/kg):I.P injected with 10 μg/kg cobra venom, group III (SV20 μg/kg): I.P. injected with 20 μg/kg cobra venom, group IV (melamine): orally 700 mg/kg melamine, group V (melamine + SV10 μg/kg): treated with 10 μg/kg cobra venom, group VI (melamine + SV20 μg/kg): treated with 20 μg/kg cobra venom. Treatment with snake venom ameliorated liver functions and increased apoptotic level marker Caspase‐3, decreased anti‐apoptotic level marker BAX. Also, decreased inflammatory level marker IL‐2 and expression level of IL‐10, INF‐γ. treatment with snake venom ameliorated hepatotoxicity induced by melamine in albino rats.

## Graphical abstract

Illegal melamine analogs are added to food to make it appear as if it contains more protein.

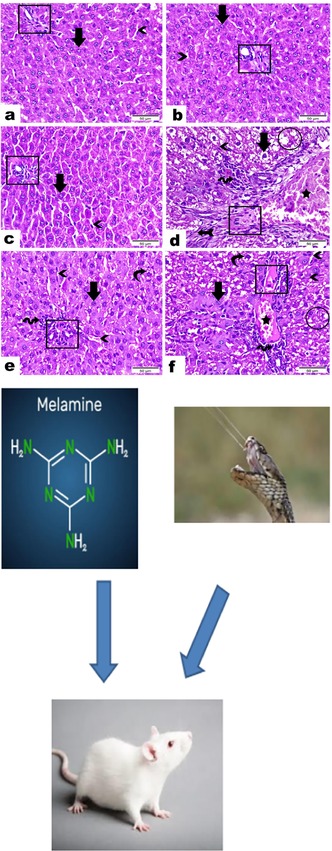

## 1 Introduction

One of the key organs in charge of the body's metabolic and excretion‐based processes is the liver. It performs a crucial role in eliminating xenobiotics and hazardous materials. Liver illnesses were thought to be the leading cause of death and morbidity worldwide, primarily as a result of the liver's inability to properly eliminate harmful substances (AlSaadi et al. 2018).

Melamine (MA) is an organic matter that is produced by synthesizing urea, a nitrogen‐based chemical. 66% of nitrogen is found in urea (Liao et al. 2021), which is produced when cyanic acid is synthesized from melamine. Melamine and formaldehyde were combined in the industry to create melamine resin, which is utilized in flame retardants, dry erase boards, textiles, adhesive materials, and long‐lasting thermosetting plastic (E.P.o.C.i.t.F. Chain, E 2010). Studies discovered that eating food polluted with melamine was the primary cause of many domestic animals' unintentional deaths (Dorne et al. 2013). Additionally, it was found that splenic lymphocytes were subjected to cytotoxic effects from MA given daily at a dose of 50 mg/kg (Wang et al. 2009).

The genus *Naja* (cobras) includes the Nubian cobra ( *Naja nubiae* ), a kind of spitting cobra that is indigenous to Africa (Theakston et al. 2003). The envenomation pattern of the Egyptian Spitting cobra is characterized by cytotoxicity (Warrell et al. 1976). Additionally, the Nubian spitting cobra has anti‐inflammatory, analgesic, and antipyretic properties that make it advantageous to use (Abdel‐Daim et al. 2015).

Cobra venom is a complex mixture primarily composed of proteins and peptides that exert various toxic effects, including neurotoxicity, tissue damage, and systemic complications. Antivenom therapy remains the primary treatment for envenomation, successfully mitigating critical effects such as bleeding and paralysis. Recent research, however, has revealed that certain venom‐derived molecules possess therapeutic potential, opening up new possibilities for drug development (Lafnoune et al. 2024). There is a growing interest in various venom components as possible sources of novel pharmaceutical substances that could be useful for treating human ailments. It has been discovered that phospholipase A2 from bee venom provides the best defense against acetaminophen‐induced hepatotoxicity (Kim et al. 2014). On the other hand, it was noted that the majority of snake venoms included two types of substances that function counter to each other by either activating or inhibiting coagulation factors (Fatima and Fatah 2014). Thus, the goal of the current research was to determine whether cobra venom could shield rats against melamine‐induced hepatotoxicity.

## 2 Materials and Methods

### 2.1 Chemicals

Melamine: Was purchased as pure from sebra chemical co., AL Asher min Ramadan city.

Cobra venom: Was purchased from the Zoology Department, Faculty of Science, Suez Canal University after it was collected by researchers in the Zoology Department, Faculty of Science, Suez Canal University.

Collection of cobra snake venom: Ten specimens of *Naja nubiae* snakes (mixture of males and females) were collected in March 2022 from the Nubian area in Aswan, Egypt, for the collection of cobra venom. The “Mirtschin technique” was the method used to collect the crude venom during the cobra's milking process (Tibballs 2001). A 70 mL bottle with a para‐film membrane wrapped over it was pushed upon the snake to bite through. Beneath the membrane, in the vial, the venom was gathered. After being combined in a 1:10 ratio with sterile distilled water, it was lyophilized (LABCONCO lyophilizer, shell freeze system, USA) and stored at −20°C.

### 2.2 Animal Management

Male adult albino rats weighing 180–200 g were acquired from the Experimental Animal Care Center at Zagazig University and accommodated in cages at the experimental animal house of the faculty of science for 7 days prior to the experiment. The environment was controlled at 25°C with a 12‐h light/dark cycle.

The guidelines for the care and use of animal subjects included in the Guide for the Care and Use of Laboratory Animals, as well as the protocol approved by the Ethics Committee (ZU‐IACUC/2/F/38/2022).

Hepatotoxicity model: For 28 days, one dose of 700 mg/kg body weight of dissolved melamine powder in warm distilled water was administered orally every day (Early et al. 2013).

### 2.3 Toxicity Study

Using 10 albino rat animals, an approximate method was used to determine the median lethal dose (LD_50_) of cobra venom (Meier and Theakston 1986).

### 2.4 Experimental Design

In order to achieve the study's ultimate objective, 48 adult male albino rats were divided into six groups, each consisted of eight animals, after they was acclimated for 7 days on a typical basal diet.

Group I (negative control group): Rats I.P. injected with 2 mL distilled water daily. Group II (Sv10 μg/kg): Rats I.P. injected with 2 mL of 10 μg/kg cobra venom once daily for 6 days. Group III (Sv20 μg/kg): Rats I.P. injected with 2 mL of 20 μg/kg cobra venom once daily for 6 days. Group IV (melamine): Rats were received (700 mg/kg B.wt.) orally day after day for 28 days. Group V (melamine + SV10 μg/kg): rats induced by melamine day after day for 28 days then intraperitoneally injected with 2 mL SV10 μg/kg for 6 days. Group VI (melamine + SV20 μg/kg): rats induced by melamine day after day for 28 days then intraperitoneally injected with 2 mL SV20 μg/kg for 6 days.

### 2.5 Blood Collection and Sampling

The rats were given a 12 h fast at the conclusion of the study, and after their final treatment, blood samples were taken from their retroorbital venous plexus while they were under a light ether anesthesia (Waynforth 1980). Two tubes were used to collect the blood samples: one for CBC analysis (including EDTA) and another for serum. Following a 20 min centrifugation at 4000*g*, serum were transferred into Eppendorf tubes and stored frozen at −20°C until biochemical tests were conducted.

### 2.6 Tissue Sample

The first part of the liver tissue samples were homogenized with ice‐cold phosphate‐buffered saline (pH 7.4) in order to prepare a 10% (w/v) tissue homogenate. The second part of the liver tissue samples was used for gene studies. The third portion underwent histopathological analysis.

### 2.7 Complete Blood Count (CBC)

Calculating the number of platelets (PLTs), white blood cells (WBCs), red blood cells (RBCs), and hemoglobin (Hb%), (MCHC), (MCV), (PDW), and (MCH) using a heparinized blood sample collected in EDTA tubes. Moreover, a differential count was carried out using an ABC vet animal blood counter for polymorph cells and lymphocytes.

### 2.8 Liver Function Tests

The assay colorimetric kit was used to measure the activities of serum ALT (alanine aminotransferase) and AST (Aspartate aminotransferase) (Reitman and Frankel 1957). The modified Bromcresol Green Binding Assay (BCG) for determination of the concentration of serum albumin (Doumas 1971). The colorimetric estimation of the total protein content is based on the idea that in alkaline circumstances, modified copper combines with protein peptide bonds to generate the distinctive pink to purple biuret complex (Doumas et al. 1981). Serum total and direct bilirubin concentrations were assessed using photometric measurement after bilirubin was converted to colored diazotized sulfanilic acid (Jendrassik and Grof 1938).

### 2.9 Determination of Rat IL‐2, BaX, and Caspase‐3 Concentration in Liver Tissue Homogenate

Levels of IL‐2, BaX, and caspase‐3 in liver tissue homogenate were determined by the ELISA technique using kits purchased from MBS biosciences Inc. (Catalog No: MBS175774) (Hollander et al. 1998), LSBIO life span bio sciences Inc. (Catalog No: LS‐F21494). MBS bio sciences Inc. (Catalog Number: MBS7244630) respectively.

### 2.10 Determination of Expression Levels of IL‐10 and INF‐γ in Liver Tissue

Following the collection of a blood sample, the tissue was preserved and frozen at −80°C. Real‐time PCR was performed.

#### 2.10.1 RNA Extraction

RNA was extracted from tissue samples sing QI Aamp R Neasy Mini kit (Qiagen, Germany, GmbH) (Bare et al. 2018).

#### 2.10.2 Oligonucleotide Primers

The primers that were used are listed in table (C) and were provided by Metabion (Germany). SYBR green One‐Step qRT‐PCR Super Mix (Trans Script Green). One microliter (μL) of each primer (forward and reverse) at a concentration of 20 pmol, 0.5 μL of reference dye, 4 μL of water, 5 μL of RNA template, and 1 μL of the 2× perfect StartTM Green One‐step qPCR Master Mix comprised the 25 mL reaction. Using a One‐Step real‐time PCR apparatus, the reaction was carried out. The thermal profile was as follows, per the kit instructions: 40 cycles of amplification (94°C for 5 s, 58°C for 15 s, and 72°C for 10 s) are performed after the reverse transcription step at 45°C for 5 min and primary denaturation at 94°C for 30 s (Batusic et al. 2011).

#### 2.10.3 Analysis of the SYBR Green RT‐PCR Results

Amplification curves and Ct values were determined by the step one software. To estimate the variation of gene expression in the RNA of the different samples, the CT of each sample was compared with that of the positive control group according to the “ΔΔCt” method stated by using the following ratio: (2^−ΔΔct^) (Bancroft and Stevens 2016).

**Table:** 

Target gene	Primers sequences 5′‐3′	Reference
B‐Actin	GGGAAATCGTGCGTGAC	Gu et al. (2021)
	AGGCTGGAAAAGAGCCT	
IL‐10	ATAAAAGGGGGACACCGGGC	Bare et al. (2018)
	CTCATAACCCATGGCTTGGC	
INFγ	GCCCTCTCTGGCTGTTACTG	Batusic et al. (2011)
	CCAAGAGGAGGCTCTTTCCT	

### 2.11 Histopathological Examination

According to the process for histological preparations involved slicing liver tissues to a thickness of 3–4 mm, fixing them in 10% neutral buffered formalin (10% NBF), dehydrating them in varying ethanol concentrations, clearing them in xylene, and embedding them in paraffin were involved. In order to study general tissue structure, the paraffin blocks were sectioned using a microtome at a thickness of 4–6 μm and then stained with hematoxylin and eosin. A Leica microscope (CH9435 Hee56rbrugg) was used to examine H&E‐stained sections (Leica Microsystems, Switzerland) (Bancroft and Stevens 2016).

### 2.12 Statistical Analysis

The mean ± SEM was used to express each parameter. One‐way ANOVA was used to statistically assess the data, and Duncan's multiple comparison test was used afterward. A *p*‐value of 0.05 was required for significant results. The SPSS 20 software package (Analytical Software, USA) was used to complete the analysis. Utilizing GraphPad Prism 8 (GraphPad, CA, USA), the charts were created.

## 3 Results

### 3.1 Toxicity Studies

It was found that the LD_50_ of snake venom is equal to 0.2 mg/kg body weight.

### 3.2 Effect of Snake Venom Treatment on Hematological Studies

The findings presented in Table 1 demonstrated a significant drop in the mean of HB level, RBC counts, MCH (Mean corpuscular hemoglobin), MCV (Mean corpuscular volume), and MCHC (Mean corpuscular hemoglobin concentration) values and a significant increase in PLT and WBC count in the melamine group compared to the control group. In treated groups (melamine + SV10 μg/kg) and (melamine + SV20 μg/kg) reported good enhancement in all parameters in compared to control group.

**TABLE 1:** Effect of different treatments on complete blood count (CBC) in all studied groups.

Groups	Negative control	SV (10/μg/kg)	SV (20/μg/kg)	Melamine	Melamine + SV (10/μg/kg)	Melamine + SV (20/μg/kg)	*p*
HB (g/dL)	13.72 ± 0.27^c^	12.87 ± 0.27^c^ −6.1%	13.67 ± 0.47^c^ −0.36%	11.72 ± 0.28*** −14.5%	12.7 ± 0.27*^a^ −7.4%	11.96 ± 0.08** −12.8	0.001
RBC (*10^6^ cell/mm^3^)	7.23 ± 0.07^c^	6.60 ± 0.21*^a^ −8.7%	6.64 ± 0.29*^a^ −8.1%	6.03 ± 0.11*** −16.5%	6.36 ± 0.15* −12%	6.15 ± 0.18** −14.9%	0.005
HCT (%)	39 ± 0.57^b^	37.42 ± 0.36^b^ −4%	35.55 ± 0.84 −8.8%	32.75 ± 0.09** −16%	36.57 ± 1.4 −6.2%	34.6 ± 1.05 −11.2%	0.001
MCV (fl)	62.07 ± 0.98^c^	58.1 ± 0.65*^a^ −6.4%	56.6 ± 0.83** −8.8%	53.45 ± 1.2*** −13.8%	56.75 ± 1.8** −8.5%	54.2 ± 0.40*** −12.6%	0.001
MCH (pg)	20.67 ± 0.49^b^	20.25 ± 0.68^a^ −2%	20.2 ± 0.44^a^ −2.2%	18.5 ± 0.30** −10.5%	19.15 ± 0.20* −7.3%	18.96 ± 0.34* −8.2%	0.019
MCHC (%)	35.37 ± 0.75^b^	34.55 ± 0.44^b^ −2.3%	35.72 ± 0.43^b^ 0.9%	33.5 ± 0.23** −5.3%	35.97 ± 0.26^b^ 1.7%	35.08 ± 0.08^a^ −0.8%	0.01
RDW (%)	20.1 ± 0.49	21.1 ± 0.23 4.9%	20.4 ± 0.49 1.5%	18.72 ± 0.31* −6.8%	19.97 ± 0.02 −0.6%	18.73 ± 0.17* −6.8%	0.002
PLT (*10^3^ cell/mm^3^	712.75 ± 14.3^c^	800.75 ± 5.7**^c^ 12.3%	877 ± 23.6***^c^ 23%	946.25 ± 26.11*** 36.9%	873.5 ± 7.6***^b^ 22.5%	915 ± 5.3*** 28.3%	0.000
WBC (*10^3^ cell/mm^3^)	10.65 ± 0.06^c^	10.8 ± 0.36^c^ 1.4%	12.32 ± 0.22** 15.6%	13.27 ± 0.35*** 24.6%	11.3 ± 0.24 ^b^ 6.1%	12.06 ± 0.14*^a^ 13.2%	0.001
Neut (%)	23.95 ± 0.46^c^	22.4 ± 0.60^b^ −6.5%	22.65 ± 0.48^c^ −5.4%	13.67 ± 0.22*** −42.9%	20.35 ± 2.3^b^ −15%	15.5 ± 3.01 −35.3%	0.001
Ly (%)	64.85 ± 0.67^c^	61.3 ± 1.3 ^c^ −5.5%	65.02 ± 1.8 ^c^ 0.26%	71.35 ± 2.7** 10%	66.37 ± 0.19 ^a^ 2.3%	69.46 ± 0.33 7.1%	0.005
Mo (%)	10.7 ± 0.23^c^	10.3 ± 0.13^c^ −3.7%	10.37 ± 0.17^c^ −3%	8.5 ± 0.23*** −20.5%	9.9 ± 0.19*^c^ −7.5%	8.73 ± 0.26*** −18.4%	0.000

*Note:* *p < 0.05 compared to control group, **p < 0.01, ***p < 0.001 compared to control group. ^a^ *p* < 0.05, ^b^ *p* < 0.01, ^c^ *p* < 0.001 compared to the positive control group. The mean difference is significant at *p* < 0.05. % change = Percent of change compared to the control group.

### 3.3 Effect of Snake Venom Treatment on Liver Function Tests

Table 2 showed a significant increase in the mean of ALT, AST activity (*p* < 0.001), T. Bil (*p* < 0.01) level, and a decrease in ALB (*p* < 0.01) & T. protein (*p* > 0.05) concentration in the melamine group compared to the control group. In snake venom‐treated groups showed good improvement in compared to the control group.

**TABLE 2:** Effect of different treatments on liver function tests in all studied groups.

Groups	Negative control	SV (10/μg/kg)	SV (20/μg/kg)	Melamine	Melamine + SV (10/μg/kg)	Melamine + SV (20/μg/kg)	*p*
ALT (U/L)	46 ± 1.08^c^	46.5 ± 0.64^c^ 1.08%	49.5 ± 2.3^c^ 7.6%	73.25 ± 4.9*** 59.2%	43.25 ± 1.4^c^ −5,9%	44.66 ± 0.88^c^ −2.9%	0.000
AST (U/L)	137.75 ± 3.8 ^c^	147.75 ± 1.72* ^c^ 7.25%	122.25 ± 4.2 ^c^ −11.25%	173.5 ± 6.5*** 25.9%	131.25 ± 1.49 ^c^ −4.7%	133 ± 4.9 ^c^ −3.4%	0.000
ALB (g/dl)	3.25 ± 0.02 ^b^	3.025 ± 0.04* −6.9%	3.125 ± 0.11 ^a^ −3.8%	2.9 ± 0.07** −10.7%	3.25 ± 0.088 ^b^ 0	3.23 ± 0.088 ^a^ 0.6	0.02
TP (g/dl)	6.45 ± 0.06	6.775 ± 0.13 ^b^ 5%	6.825 ± 0.21 ^b^ 5.8%	6.075 ± 0.26 −5.8%	6.55 ± 0.02 ^a^ 1.5%	6.2 ± 0.11 −3.8%	0.02
T.Bil	0.36 ± 0.015 ^b^	0.38 ± 0.026 ^a^ 5.5%	0.47 ± 0.015* ^b^ 30.5%	0.61 ± 0.006** 69.4%	0.47 ± 0.010* ^b^ 30.5%	0.51 ± 0.008** ^b^ 41.6%	0.000
Direct. Bil (mg/dl)	0.102 ± 0.002	0.102 ± 0.002 0%	0.135 ± 0.018 32.3%	0.130 ± 0.004 27.4%	0.125 ± 0.002 22.5%	0.12 ± 0.005 17.6%	0.05

*Note:* *p < 0.05 compared to control group, **p < 0.01, ***p < 0.001 compared to control group. ^a^ *p* < 0.05, ^b^ *p* < 0.01, ^c^ *p* < 0.001 compared to the positive control group. The mean difference is significant at *p* < 0.05. % change = Percent of change compared to the control group.

### 3.4 Effect of Snake Venom Treatment on IL‐2, BAX, and Caspase‐3 Level in Liver Tissue of Studied Groups

**FIGURE 1:**
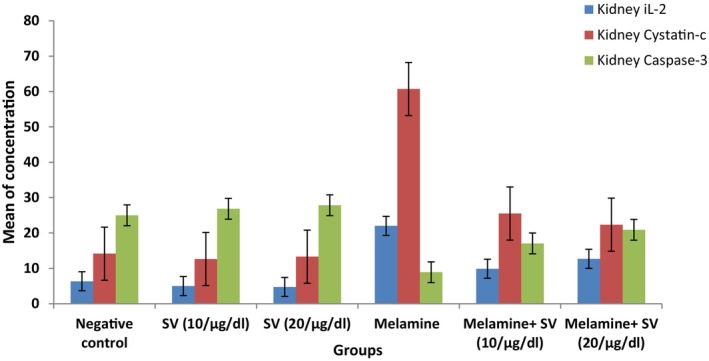
Mean of inflammatory, apoptotic, and anti‐apoptotic markers in all studied groups.

The findings presented in Table 3; Figure 1 indicated a noteworthy rise in the mean IL‐2 & BAX level in the melamine group, amounting to 249.6% & 191.5% respectively (*p* < 0.01) when compared to the control group. Also, the mean Caspase‐3 level in the melamine group dropped significantly by −64% (*p* < 0.01) in comparison to the control group.

**TABLE 3:** Effect of different treatments on inflammatory, apoptotic, and anti‐apoptotic markers in liver tissues of all studied groups.

Groups	Negative control	SV (10/μg/kg)	SV (20/μg/kg)	Melamine	Melamine + SV (10/μg/kg)	Melamine + SV (20/μg/kg)	*p*
Liver iL‐2 (ng/g tissue)	8.05 ± 0.77^b^	6.35 ± 0.25^b^ −21.1%	7.05 ± 0.08^b^ −12.4%	28.15 ± 1.7** 249.6%	12.35 ± 0.6^a^ 53.4%	15.7 ± 0.28**^a^ 95.03%	0.000
Liver BAX (ng/g tissue)	10.7 ± 0.51^b^	7.1 ± 0.4^b^ −33.6%	8.8 ± 0.22^b^ −17.7%	31.2 ± 1.67** 191.5%	14.2 ± 0.57*^b^ 32.7%	18.8 ± 0.92**^a^ 75.7%	0.000
Liver Caspase‐3 (ng/g tissue)	27.8 ± 1.31^b^	30 ± 1.34^b^ 7.9%	30.2 ± 2.31^a^ 8.6%	10 ± 0.71** −64%	17.7 ± 0.86*^b^ −36.3%	23.2 ± 0.63^c^ −16.5%	0.000

*Note:* *p < 0.05 compared to control group, **p < 0.01, ***p < 0.001 compared to control group. ^a^ *p* < 0.05, ^b^ *p* < 0.01, ^c^ *p* < 0.001 compared to the positive control group. The mean difference is significant at *p* < 0.05. % change = Percent of change compared to the control group.

### 3.5 Effect of Snake Venom Treatment on Gene Expression Level of IL‐10 and INF‐γ in Tissue of Liver of Studied Groups

**FIGURE 2:**
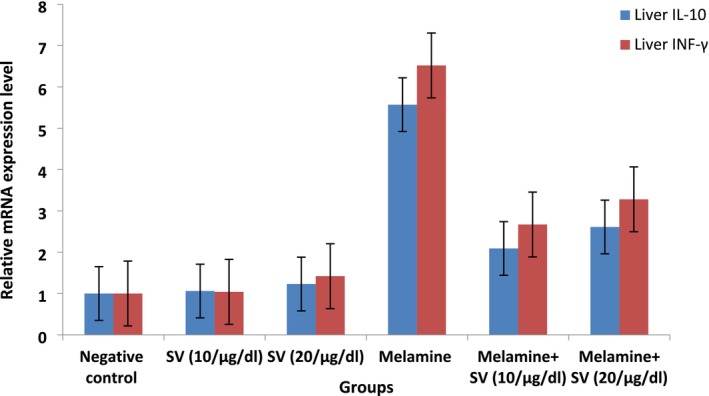
Mean Relative mRNA expression level of il‐10 and INF‐γ in liver tissue of all studied groups.

The findings presented in Table 4; Figure 2 indicated a high rise in the mean of IL‐2 and INF‐γ expression levels in the melamine group, amounting to 457% and 552%, respectively (*p* < 0.001) when compared to the control group.

**TABLE 4:** Relative mRNA expression level of IL‐10 and INF‐γ in liver tissues of all studied groups.

Groups	Negative Control	SV (10/μg/kg)	SV (20/μg/kg)	Melamine	Melamine + SV (10/μg/kg)	Melamine + SV (20/μg/kg)	*p*
Gene expression of IL‐10	1 ± 0.00^c^	1.06 ± 0.006***^c^ 6%	1.23 ± 0.007***^c^ 23%	5.57 ± 0.04*** 457%	2.09 ± 0.004***^c^ 109%	2.61 ± 0.02***^c^ 161%	0.000
Gene expression of INF‐γ	1 ± 0.00^c^	1.04 ± 0.012^c^ 4%	1.42 ± 0.008***^c^ 42%	6.52 ± 0.013*** 552%	2.67 ± 0.04***^c^ 167%	3.28 ± 0.005***^c^ 228%	0.000

*Note:* *p < 0.05 compared to the control group, **p < 0.01, ***p < 0.001 compared to the control group. ^a^ *p* < 0.05, ^b^ *p* < 0.01, ^c^ *p* < 0.001 compared to the positive control group. The mean difference is significant at *p* < 0.05. % change = Percent change compared to the control group.

### 3.6 Histopathological Examination

**FIGURE 3:**
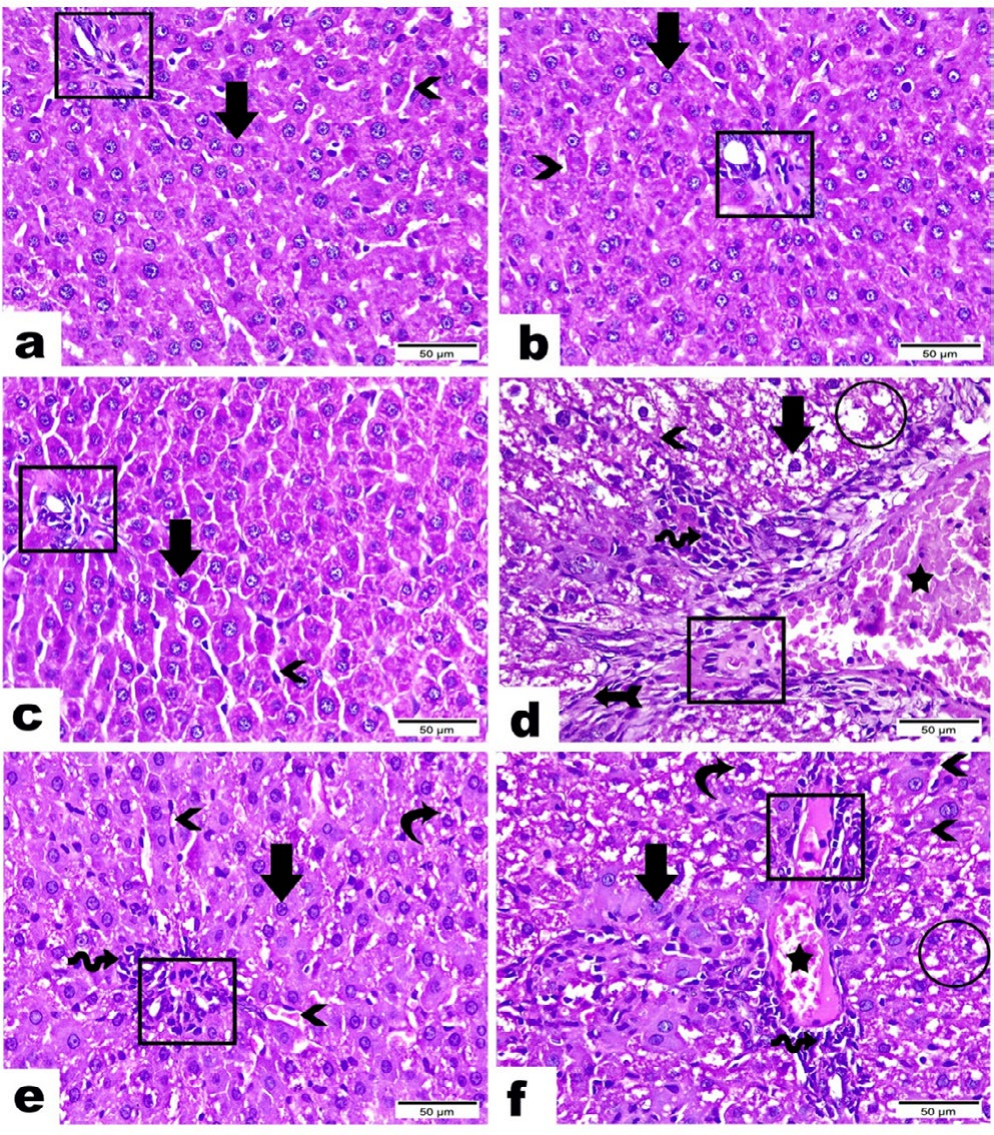
Photomicrographs displaying the histopathological differences in liver tissue sections (Portal Area) between tested groups (Hematoxylin & Eosin Stain, Magnification Power = *x*400 & Scale Bar = 50 μm) as follows: Group I: Negative Control, GroupII: SV10 μg/dL, and GroupIII: SV20 μg/dl demonstrating the normal architecture of the portal triad with intact portal vein, hepatic artery, and bile duct (rectangles). Hepatic cords are organized in a regular look containing large hepatocytes with central, spherical, and vesicular nuclei (arrows). Notice hepatic sinusoids disclosing between hepatic cords (arrowheads). GroupIV: Melamine highlighting serious degenerative changes including severe dilatation, congestion (star) and hyalinization (rectangle) of the portal vein, an obvious increase in fiber amount (arrow with tail), excessive aggregation of inflammatory cells (wave arrow), loss of hepatic organization (circle), hydro pic degeneration of hepatocytes (arrow), and atrophy along hepatic sinusoids (arrowhead). Group V: Melamine + SV 10 μg/dL revealing a marked improvement in the tissue architecture manifested by few congestions along the portal vein (rectangle), regular hepatic cords with nearly normal hepatocytes (arrow) except few denoted with mild hepatocellular degeneration (curvy arrow), few aggregations of inflammatory cells (wave arrow), in addition to most blood sinusoids marked in intact look while few noticed with congestion (arrowheads). Group VI: Melamine + SV 20 μg/dL displaying a few recoveries evidenced by most areas still marked with loss of its organization (circle), few apparently normal hepatocytes (arrow) while most hepatocytes disclosed with hydropic degeneration (curvy arrow), moderate dilatation, congestion (star), and hyalinization of the portal vein (rectangle), moderate aggregation of inflammatory cells (wave arrow), as well as few intact hepatic sinusoids and the majority demonstrated in an atrophied appearance (arrowheads).

Group I: (Negative Control), Group II: (SV10 μg/kg), and Group III: (SV20  μg/kg) demonstrating the normal architecture of the portal triad with intact portal vein, hepatic artery, and bile duct (rectangles). Hepatic cords are organized in a regular look containing large hepatocytes with central, spherical, and vesicular nuclei (arrows). Notice hepatic sinusoids disclosing between hepatic cords (arrowheads). Group IV:(Melamine Group) highlighting serious degenerative changes including severe dilatation, congestion (star) and hyalinization (rectangle) of the portal vein, an obvious increase in fiber amount (arrow with tail), excessive aggregation of inflammatory cells (wave arrow), loss of hepatic organization (circle), hydropic degeneration of hepatocytes (arrow), and atrophy along hepatic sinusoids (arrowhead). Group V: (Melamine + SV10 μg/kg) reveals a significant change in the tissue architecture as seen by the regular hepatic cords with almost normal hepatocytes (arrow), and the few congestions along the portal vein (rectangle) except for a few denoted with mild hepatocellular degeneration (curvy arrow), few aggregations of inflammatory cells (wave arrow), in addition to most blood sinusoids marked in an intact look while a few are noticed with congestion (arrowheads). Group VI (Melamine + SV20 μg/kg) displaying a few recovery evidenced by most areas still marked with a loss of their organization (circle), a few apparently normal hepatocytes (arrow) while most hepatocytes disclosed with hydropic degeneration (curvy arrow), moderate dilatation, congestion (star), and hyalinization of the portal vein (rectangle), moderate aggregation of inflammatory cells (wave arrow), as well as a few intact hepatic sinusoids and majority are demonstrated in an atrophied appearance (arrowheads) Figure 3.

## 4 Discussion

Chemical‐induced hepatotoxicity depends on multiple factors, such as the toxicant concentration, the specific expression of enzymes, and the gradient of the substance's concentration in the blood that surrounds the acinus (Pervez 2020). Melamine showed toxic effects on liver tissue (Erisgin et al. 2021). It has been observed that melamine, either alone or in conjunction with cyanuric acid, can cause pathologic liver lesions in mice (Erisgin et al. 2021).

It was found that cobroxin and nyloxin, two analgesics, are present in Egyptian cobra venom. Nyloxin is useful in treating severe arthritis pain, while cobroxin works similarly to morphine by blocking nerve transmission (Chang et al. 2021). Effectiveness as an anticoagulant has been shown for the Arvin compound, which is extracted from the Malayan pit viper. Enzymes called phospholipase A_2_ are capable of hydrolyzing phospholipids, which may be directed towards the surfaces of bacterial cells and possess novel antimicrobial qualities (Estevão‐Costa et al. 2018). Many of the proteins found in snake venom have long been known to have analgesic qualities (Bocian and Hus 2020); the main challenge is getting the protein into the nerve cells efficiently. Also used in the treatment of bone and breast cancer (Mohamed Abd El‐Aziz 2019).

In our results, melamine significantly decreased the mean HB level, RBC count, neutrophil count, and monocyte count, while PLT count, WBC count, and lymphocyte cells were significantly elevated in comparison to control groups. These findings ensured the findings of the researchers who found that, in comparison to the control, melamine significantly decreased RBC, HB, HCT, MCV, and MCHC values; thus, the exposure to melamine affects the body's hematopoietic function and caused increase in WBC counts that reflects the inflammatory status (Liu et al. 2022). Also, our findings corroborated those of other researchers who found that melamine significantly reduced the mean values of RBCs, Hb, and MCHC in the melamine group when compared to the control groups and may increase the risk of RBC abnormality (Abd‐Elhakim et al. 2016).

Melamine (MEL) may be linked to aberrant protein expression and cell membrane rupture in relation to standard blood parameters. Studies declared that MEL causes membrane osmotic resistance and Na^+^/K^+^ATPase activity to diminish, weakening cell membrane integrity and potentially rupturing blood cell membranes (Strakova et al. 2014).

The application of cobra venom resulted in increased RBC, HB, total WBC, and MCHC counts, as well as decreased neutrophil and monocyte counts.

The present study suggests that exposure to melamine may have facilitated the circulation's release of the liver enzymes AST, ALT, D, and T bilirubin, leading to elevated serum levels. These findings support previous reports (Aboubakr et al. 2021; Ahmed et al. 2021; Ahmed et al. 2022) that showed increased hepatic enzyme levels. Additionally, there was also a report of notable hepatocellular membrane degradation accompanied by elevated hepatic enzyme activities, confirming a significant impairment of liver function following melamine exposure (El Rabey et al. 2014; Early et al. 2013). These results are consistent with our histoarchitecture data, which showed significant pathologic changes in liver tissues, including significant hepatocyte membrane damage.

Furthermore, after being exposed to melamine, researchers noticed the hepatic structure being disrupted and hepatic aggregates formed (Melekoğlu et al. 2020). Previous studies have also reported on this observation (Chang et al. 2021; Ahmed et al. 2022) proving beyond a doubt that melamine damages rat liver tissue. The current study's findings demonstrated the therapeutic efficacy of administering Egyptian cobra venom against melamine‐induced hepatic dysfunction by increasing the activity of T. protein and albumin and decreasing the activities of AST, ALT, T. bilirubin, and D. bilirubin.

Through substrate inactivation and the production of active signaling molecules, the caspase family of endoproteases induces inflammation and apoptosis (He et al. 2021). Both intrinsic (mediated by mitochondria) and external (external) cell death are generally involved in cell apoptosis (Yang et al. 2022). Caspase‐3 is a crucial executioner caspase in apoptosis. Once activated, it cleaves various cellular substrates, leading to controlled cell dismantling and death. Once activated, caspase‐3 cleaves multiple substrates, leading to Cellular breakdown: Cleavage of PARP (Poly ADP‐Ribose Polymerase) → Prevents DNA repair, Degradation of ICAD (Inhibitor of Caspase‐Activated DNase) → Releases CAD (Caspase‐Activated DNase), leading to DNA fragmentation and Cytoskeletal and nuclear breakdown → Cleavage of actin, lamin, and gelsolin disrupts cell structure.

These events result in membrane blebbing, chromatin condensation, and apoptotic body formation, leading to phagocytic clearance. Conversely, a number of cellular stressors, including UV and oxidative stress, can trigger intrinsic cell apoptosis by negatively regulating Bcl2, increasing the translocation of Bax to the mitochondria, and causing damage to the mitochondrial membrane. Bax is a key pro‐apoptotic member of the Bcl‐2 family involved in mitochondrial‐mediated apoptosis. Under normal conditions, Bax is mainly located in the cytosol in an inactive monomeric form, bound to anti‐apoptotic proteins like Bcl‐2 and Bcl‐xL. Upon receiving pro‐apoptotic signals (e.g., DNA damage, growth factor withdrawal, oxidative stress), Bax undergoes a conformational change and translocates to the outer mitochondrial membrane (OMM). This activation is triggered by BH3‐only proteins like Bid, Bim, or Puma, which release Bax from inhibitory interactions. Once at the mitochondrial membrane, Bax undergoes oligomerization and inserts into the OMM. This leads to mitochondrial outer membrane permeabilization (MOMP), a key step in apoptosis. Bax forms pores or channels in the membrane, allowing the release of cytochrome c and other pro‐apoptotic factors. Cytochrome c is released from mitochondria into the cytoplasm. It binds to Apaf‐1 (apoptotic protease activating factor‐1), forming the apoptosome. The apoptosome activates caspase‐9, which in turn activates caspase‐3 and caspase‐7, leading to cellular dismantling and apoptosis (Yang et al. 2022). The present investigation's findings suggest that melamine‐induced hepatotoxicity is linked to both intrinsic and extrinsic hepatic apoptosis. This is supported by the observed rise in Bax transcription, decrease in caspase‐3, an anti‐apoptotic enzyme, and concurrent decrease in Bcl2 mRNA levels. Other authors also demonstrated similar results, confirming that intrinsic cell death (Habotta, Ateya, et al. 2023; Alsharif et al. 2023).

The level of hepatotoxicity in comparison to the control group was evaluated by assessing apoptosis, non‐apoptosis markers BAX, and caspase‐3, based on the obtained findings. When compared to the control group, the BAX level was significantly higher in the melamine group (Knittel et al. 1999). In this investigation, the treated groups' BAX levels were lower than those of the melamine group. In addition, the treated group's level of active Caspase‐3 was higher than the melamine group's. When a protein antigen enters the body, the immune response—a highly complex and regulated process—is set off. The liver was once thought of as a non‐immunological organ that was mainly used for metabolic, nutritional storage, and detoxification functions. Nonetheless, new discoveries have revealed that the healthy liver is also the location of intricate immune activity, which is controlled by a diverse range of immune cells that are in charge of both inflammatory and anti‐inflammatory responses (Strakova et al. 2014; Aboubakr et al. 2021).

Anti‐apoptotic and inflammatory markers were elevated in melamine (Robinson et al. 2016). Increased production of pro‐inflammatory cytokines and important inflammatory cell mechanisms involved in melamine hepatotoxicity have been linked to hepatic injuries caused by methamine (Yang et al. 2022; Habotta, Ateya, et al. 2023). This result is consistent with both our immunohistochemistry results, which showed elevated expression of an inflammatory marker in liver tissue, and our histopathological results, which explain inflammatory cell infiltration. The intriguing transcription factor known as an inflammatory marker is involved in the regulation of pro‐inflammatory mediators, chemokine expression, and cytokine activation (Habotta, Abdeen, et al. 2023). Similar findings have been reported regarding the upregulation of inflammatory expression in rats that have been given melanin (Yang et al. 2022). In this study, the treated group showed higher concentrations of inflammatory markers like IL‐2, IL‐10, and INF‐γ, indicating improvements in liver tissue. Highly anti‐inflammatory cytokines like IL‐2, IL‐10, and IFN‐ℽ were present in the hepatotoxicity melamine group in this experiment, but they decreased following treatment with Egyptian cobra venom. Increased levels of IL‐2 and IL‐10 significantly increase T‐lymphocyte activation in hepatotoxicity (Ahmed et al. 2022). Interleukin‐2 (IL‐2) is a cytokine that plays a crucial role in immune system regulation, particularly in T‐cell proliferation, survival, and differentiation. IL‐2 is primarily produced by activated CD4^+^ T cells, though CD8^+^ T cells and natural killer (NK) cells can also produce it. Its expression is triggered by antigen stimulation via the T‐cell receptor (TCR) and costimulatory signals, mainly CD28. Transcription of IL‐2 is regulated by transcription factors such as NF‐κB, AP‐1, and NFAT. Once IL‐2 binds to its high‐affinity receptor (IL‐2Rα/β/γc), it triggers intracellular signaling via JAK–STAT, PI3K‐Akt, and MAPK pathways which lead toT‐cell proliferation, regulatory T cell (Treg) maintenance that is essential for immune tolerance and prevention of autoimmunity, memory T‐cell formation that supports long‐term immune responses, and cytotoxic T cell (CTL) and NK cell activation.

Interleukin‐10 (IL‐10) is an anti‐inflammatory cytokine that plays a key role in immune regulation, inflammation suppression, and tissue homeostasis. It is produced mainly by regulatory T cells (Tregs), monocytes, macrophages, and B cells. IL‐10 inhibits the production of TNF‐α, IL‐6, IL‐12, and IFN‐γ by macrophages and dendritic cells. It downregulates NF‐κB, preventing inflammatory gene transcription. Additionally, IL‐10 enhances Foxp3+ Treg cell differentiation. Tregs suppress excessive immune activation, preventing autoimmunity. Therefore, IL‐10 limits excessive immune damage in infections and chronic inflammation and supports tissue repair and resolution of inflammation.

Interferon‐gamma (IFN‐γ) is a pro‐inflammatory cytokine that plays a crucial role in immune responses against infections, tumor immunity, and autoimmune regulation. It is primarily produced by T cells (CD4^+^ Th1 and CD8^+^ cytotoxic T cells) and natural killer (NK) cells. IFN‐γ binds to the IFN‐γ receptor (IFNGR), which is expressed on nearly all nucleated cells, especially macrophages, dendritic cells (DCs), and epithelial cells, leading to JAK–STAT Activation that induces ROS and NO production, increases microbial killing, and upregulates MHC Class II and CD80/CD86, boosting T‐cell activation. This is a multi‐level process that may also be partially dependent on the local synthesis of chemoattractant anti‐cytokines (IFN‐γ) or chemokines, which control the function of cell‐surface adhesion receptors and guide the migration of targeted cells into the tissue site (Fatima and Fatah 2014). Furthermore, increased expression of pro‐inflammatory chemokine genes was noted in cases of liver damage (Doumas et al. 1981). In the current investigation, rats were given melamine to cause irreversible liver damage. Liver damage was indicated by elevated levels of acute‐phase cytokines, such as IFN‐γ, IL‐2, and IL‐10, as reported in studies (Erisgin et al. 2021). Liver enzymes, pro‐inflammatory cytokines, and inflammatory markers were all balanced in those receiving treatment from Egyptian cobra venom. The current study's findings regarding histopathological alterations revealed that rats given melamine displayed a variety of hepatocyte degenerative changes, including enlarged cells. The cells had a foamy, light appearance. Vacuoles, or multiple spaces occupied by cells, were seen in the cytoplasm. Blood vessel dilatation also caused necrosis in some liver cells that had condensed chromatin and small, psychotic nuclei. The outcomes corroborated the findings of (Pervez 2020), who stated that melamine resulted in massive lymphocyte infiltration, necrotic changes, hepatic tissue degeneration, and significant fatty changes. Furthermore, that (Abd‐Elhakim et al. 2016). Conversely, there were less necrotic alterations and liver tissue degeneration in the treated groups.

## 5 Conclusion

In the treatment of melamine‐induced hepatotoxicity, treatment with (melamine + SV10 ug/kg) had a protective effect. It can be inferred from the current experimental data that rats' protected liver tissue can withstand a concentration of cobra venom (SV10 μg/kg) more safely than SV20 μg/kg.

## Author Contributions

**Al‐Shimaa M. Abas:** conceptualization (equal), funding acquisition (equal), supervision (equal), writing – review and editing (equal). **Akaber T. Keshta:** writing – original draft (equal). **Samah A. Mohammed:** methodology (equal), writing – original draft (equal). **Shimaa H. Watad:** conceptualization (equal), funding acquisition (equal), supervision (equal), writing – review and editing (equal).

## Conflicts of Interest

The authors declare no conflicts of interest.

## Data Availability

The authors confirm that the data supporting the findings of this study are available within the article.
